# Systematic analysis of the UDP-glucosyltransferase family: discovery of a member involved in rutin biosynthesis in *Solanum melongena*

**DOI:** 10.3389/fpls.2023.1310080

**Published:** 2023-12-22

**Authors:** Yuwei Gan, Bingwei Yu, Renjian Liu, Bingbing Shu, Yonggui Liang, Yafei Zhao, Zhengkun Qiu, Shuangshuang Yan, Bihao Cao

**Affiliations:** Key Laboratory of Biology and Genetic Improvement of Horticultural Crops (South China), Ministry of Agriculture and Rural Affairs/Guangdong Vegetable Engineering and Technology Research Center/College of Horticulture, South China Agricultural University, Guangzhou, China

**Keywords:** eggplant, UGT superfamily, rutin, biosynthesis, functional identification

## Abstract

Eggplant (*Solanum melongena*) is an economically important crop and rich in various nutrients, among which rutin that has positive effects on human health is found in eggplant. Glycosylation mediated by UDP-glycosyltransferases (UGTs) is a key step in rutin biosynthesis. However, the *UGT* gene has not been reported in eggplant to date. Herein, 195 putative *UGT* genes were identified in eggplant by genome-wide analysis, and they were divided into 17 subgroups (Group A-P and Group R) according to the phylogenetic evolutionary tree. The members of Groups A, B, D, E and L were related to flavonol biosynthesis, and rutin was the typical flavonol. The expression profile showed that the transcriptional levels of *SmUGT* genes in Clusters 7-10 were closely related to those of rutin biosynthetic pathway genes. Notably, *SmUGT89B2* was classified into Cluster 7 and Group B; its expression was consistent with rutin accumulation in different tissues and different leaf stages of eggplant. SmUGT89B2 was located in the nucleus and cell membrane. Virus-induced gene silencing (VIGS) and transient overexpression assays showed that *SmUGT89B2* can promote rutin accumulation in eggplant. These findings provide new insights into the UGT genes in eggplant, indicating that SmUGT89B2 is likely to encode the final enzyme in rutin biosynthesis.

## Introduction

As an important horticultural crop, eggplant (*Solanum melongena* L.) is the third most cultivated *Solanaceae* species in the world, next to potato (*Solanum tuberosum* L.) and tomato (*Solanum lycopersicum* L.) (Barchi et al., 2021). Eggplant is planted on 1.85 million hectares with a worldwide production of 56.30 million tons, which is mainly cultivated in India and China, and consumed worldwide (FAO, see www.fao.org) (Martínez-Ispizua et al., 2021; Wang et al., 2022). Flavonoids are one of the most valued compounds in eggplant fruit: anthocyanins are the highest, followed by flavonol (Martínez-Ispizua et al., 2021). Currently, all kinds of flavonols have been identified in eggplant fruit, including quercetin, rutin, myricetin, quercetin-3-diglucoside, kaempferol-3,7-diglucoside, kaempferol-diglucoside and quercetin-3-rhamnoside (Singh et al., 2017; Sulli et al., 2021). Flavonols are plant-specialized metabolites with important functions in regulate plant development, signaling, and stress response (Daryanavard et al., 2023).

Rutin is a typical flavonol and plays a key role in defense responses against abiotic and biotic stresses, such as resistance to low temperatures, adaptation to environmental change, and tolerance to fungal and viral pathogens (Suzuki et al., 2005; Yang et al., 2022). Rutin has wide range of pharmacological activities and high antioxidant capacity. It is widely used in clinical medicine, e.g., for anticarcinogenic, antiviral and anti-inflammation treatment and for the protection of nerves and the cardiovascular system (Glasser et al., 2002; Jimenez-Aliaga et al., 2011; Luca et al., 2020). Rutin is the diglycoside form of quercetin, which is the main flavonol in vegetables and fruits. It is the most popular dietary flavonoid compound in the human diet (Hosseinzadeh and Nassiri-Asl, 2014; Tobar-Delgado et al., 2023). Although the biosynthetic pathway of flavonol is clear, the enzyme in the final two glycosylation steps of quercetin conversion into rutin remains elusive (**Figure S1A**) (Vogt, 2010; Zhang et al., 2017; Shen et al., 2022). *UGT* encodes the final enzyme in the rutin biosynthetic pathway and determines the pharmaceutical functions of rutin (Jing et al., 2016).Glycosylation is one of the most important modifications. Glycosylation catalyzed by glycosyltransferase is an important modification process in plants and is one of the final steps involved in secondary metabolite synthesis (Caputi et al., 2012). Glycosyltransferase catalyzes the transfer of glycosyl from activated donor molecules onto various small molecule receptors, leading to the production of di-saccharides, poly-glycosides, and diversified glycosides of noncarbohydrate moieties (Caputi et al., 2012; Wilson and Tian, 2019). The plant secondary product glycosyltransferase (PSPG) box is the binding site of the sugar donor. It consists of a conserved sequence of 44 amino acid residues in the UGT of plants near the C-terminus (Gachon et al., 2005; Offen et al., 2006). The UDP-glycosyltransferase superfamily is a large family that possesses redundant functions and complex structures and presents distinct but overlapping substrate specificities (Mackenzie et al., 1997). Members of the UGT superfamily are named using the acknowledged convention (**Figure S1B**). Numbers from 1 to 50 are utilized for animals, 51 to 70 for yeasts and amoebae, 71 to 100 for plants and 101 to 200 for bacteria (**Figure S1B**) (Ross et al., 2001). With the development of molecular biology, an increasing number of *UGT* genes have been cloned and characterized, which are enrich of rutin biosynthesis. Rutin is formed by the two-step glycosylation of quercetin. For example, FaGT6 was proven to be responsible for the first step, which catalyzed quercetin to 3-*O*-glucosides (Griesser et al., 2008). *UGT* genes with similar functions in the first step of glycosylation have been reported in both soybean (Kovinich et al., 2010) and grape (Mizohata et al., 2013). GmUGT79A6 was identified as rhamnose-glycosyltransferase involved in the second step of rutin glycosylation (Rojas et al., 2014). In addition, FeUGT79A8 (Koja et al., 2018) and FtUGT73BE5 (Yin et al., 2020) were reported to promote rutin accumulation in buckwheat. Many *UGT* genes involved in key secondary metabolite biosynthesis have been successfully identified and investigated in different plant species, including *Arabidopsis* (Paquette et al., 2003), pomegranate (Wilson et al., 2019), buckwheat (Yin et al., 2020), soybean (Yin et al., 2017), and tea (Cui et al., 2016; Dai et al., 2022). However, the genes of rutin biosynthesis are still unknown in eggplant.

In this study, the pangenome of eggplant was used to identify the UGT family and obtain potential *UGT* genes that might be related to rutin biosynthesis. A total of 195 *UGT* candidate genes were identified in eggplant, and their gene structure and evolutionary relationship were analyzed. Five *UGT* candidate genes were identified with rutin biosynthesis *via* expression pattern and correlation analysis. Furthermore, *SmUGT89B2* was proven to participate in rutin biosynthesis in eggplant by using VIGS and transient overexpression assays.

## Material and methods

### Identification of UGTs in the eggplant genome and their chromosomal locations

UGT families were retrieved from the eggplant genome V4.0 downloaded from the Solanaceae genomics database (https://solgenomics.net/) by using the following two methods: 44 amino acid sequences of PSPG-box were used as query sequences to blast the eggplant, and the Hidden Markov Model (HMM) profile of PF00201. The members of UGT families needed to meet the requirement of the two methods. The NCBI Conserved Domain Search Service (Batch CD-Search) and SMART were used to filter redundant sequences. The full-length amino acid sequences, molecular weight (MW), theoretical isoelectric point (PI) and instability index of the UGT proteins were predicted by the ExPASy server. Based on the gff3 file of eggplant, the distribution of *UGT* genes on chromosomes was retrieved and visualized by using TBtools (Chen et al., 2020).

### Sequence alignment and phylogenetic analysis

The full-length amino acid sequences of all SmUGTs were aligned by Clustal X2 software with default values used for all parameters. The phylogenetic evolutionary tree was constructed using the maximum likelihood method through MEGA X software with 2000 bootstrap replications (Kumar et al., 2018). The 122 UGT protein sequences of Arabidopsis used in the tree were downloaded from the site (http://www.p450.kvl.dk/index).

### Gene structure and conserved motif analysis

Based on the relationship of the coding sequence and its corresponding genomic DNA sequence from the gff3 file, the gene structure of *SmUGTs* was visualized by using TBtools. Conserved motifs or domains were searched by MEME (https://meme-suite.org/meme/tools/meme)(Bailey et al., 2015), with parameters as follows: number of motif predictions, 20; minimum motif width, 6; maximum motif width, 50 (Bailey et al., 2009), and then visualized by using TBtools.

### RNA-seq data analysis

The transcriptome data at different developmental stages of eggplant fruit were obtained from previous eggplant genome research (Barchi et al., 2019). A heatmap reflecting the expression patterns of *SmUGTs* at different developmental stages of eggplant fruit was constructed by TBtools.

### Plant materials

The E801 inbred line used in the study was provided by our lab. Eggplant seeds were sown in hole trays when the seedlings grew to the three-leaf stage and transplanted to the experimental farm (South China Agricultural University, Qilin). The different tissues of eggplant, including the roots, stems, leaves, flowers and commercial fruits, were collected, immediately frozen in liquid nitrogen and then stored at -80°C for RNA extraction and determination of rutin content. The different developmental stages of eggplant fruits at 5 DPA, 12 DPA, 15 DPA, 22 DPA and 30 DPA were collected, and immediately frozen in liquid nitrogen and then stored at -80°C for RNA extraction and determination of rutin content (**Figure S2**).

### Total RNA extraction and quantitative real-time PCR

Total RNA was extracted from eggplant tissues by an Eastep Super Total RNA Extraction Kit (Promega, WI, USA) according to Liu et al. (Liu et al., 2023). First-strand cDNA was synthesized by using a cDNA Kit PLUS (B0003) (EZBioscience, Roseville, MN, USA) according to the manufacturer’s instructions. All primer sequences are listed in **Table S2**, including reference genes. The relative expression of UGT genes was calculated as described by Song et al. (Song et al., 2020). The qPCRs using different tissues were performed with biological triplicates.

### Determination of rutin content

The rutin content was detected according to Dong et al. (Dong et al., 2020). Fresh tissues (0.1 g) were ground into a fine powder with liquid nitrogen, extracted with 2 mL ethanol (60%) and centrifuged for 10 min at 12000 rpm. The supernatant was transferred to a new 2 mL centrifuge tube and placed in a metal bath at 80 °C for 2 h. After heating, 0.4 mL of supernatant was mixed with 0.4 mL of AlCl_3_ (0.1 mol/L) and 0.6 mL of CH_3_COOK (1 mol/L) solution and then added to 0.6 mL of ethanol (60%). The mixture was shaken and allowed to stand for 30 minutes. The absorbance of the solution was measured using a microplate reader at 420 nm. Determination of rutin content was performed in triplicate.

### Subcellular localization analysis

To confirm the Subcellular localization of *SmUGT89B2*, the coding DNA sequence (CDS) of SmUGT89B2 was cloned from eggplant. Its coding sequence without a terminator codon was cloned and inserted into a vector fused with EGFP carrying the CaMV 35S promoter to generate the 35S: SmUGT89B2-EGFP construct by using one-step cloning (C112, Vazyme, China) according to the manufacturer’s instructions. The recombinant plasmid was transformed into competent cells of *Agrobacterium* strain GV3101. *Agrobacterium tumefaciens* GV3101 containing the corresponding constructs was incubated with YEP medium at 28°C. The bacterial cells were collected and modulated 0.4-0.6 at 600 nm using MS medium (10 mM 2-(4-morpholino)-ethane sulfonic acid, 10 mM MgCl_2_, and 200 μM acetosyringone, pH = 5.6) and then placed in darkness at 28°C for 2 h. *Agrobacterium tumefaciens* GV3101 containing the 35S:SmUGT89B2-EGFP construct and empty vector were mixed with the NLS-DsRed construct in equal proportions (1:1). The mixed solution was infiltrated into the abaxial side of an *Nicotiana benthamiana* leaf. Three days later, the GFP signal of leaves that were inoculated with *Agrobacterium tumefaciens* was observed by using fluorescence microscopy (Leica, Germany) (Song et al., 2023). The primers used are listed in **Table S2**.

### Virus-induced gene silencing assays

The full-length CDS of SmUGT89B2 was predicted to be a hypothetically optimal fragment (300 bp) by using the Solanaceae database (http://vigs.solgenomics.net/). The 300 bp fragment was amplified and ligated to the pTRV2 vector. pTRV2-*SmUGT89B2*, pTRV1 and pTRV2 were transformed into competent cells of Agrobacterium strain GV3101. *Agrobacterium tumefaciens* GV3101 containing pTRV2-*SmUGT89B2* and pTRV1 were mixed in equal proportions, and the bacterial solution was infiltrated into the abaxial side of 30-day-old seedlings of eggplant with a 1 mL syringe. The eggplant plants were grown in a growth chamber under growth conditions as follows: 60–70% humidity, 22-25°C, 16 h/8 h (light/dark cycle). The primers used are listed in **Table S2**.

### Transient overexpression analysis

Transient overexpression analysis was performed according to a previous method (Wang et al., 2022). The full-length coding sequence of *SmUGT89B2* was cloned and inserted into the PC1300 vector. The recombinant vector was transformed into competent cells of Agrobacterium strain GV3101. The bacterial solution containing the corresponding constructs was infiltrated into the abaxial side of eggplant (E801) leaves at the five-leaf stage, and the plants were grown in a growth chamber. After 5 days, the leaves of eggplant were collected, frozen in liquid nitrogen and stored at −80°C for RNA extraction and determination of rutin content. The primers used are listed in **Table S2**.

## Results

### Identification and chromosomal localization of eggplant UGT genes

**Figure 1:**
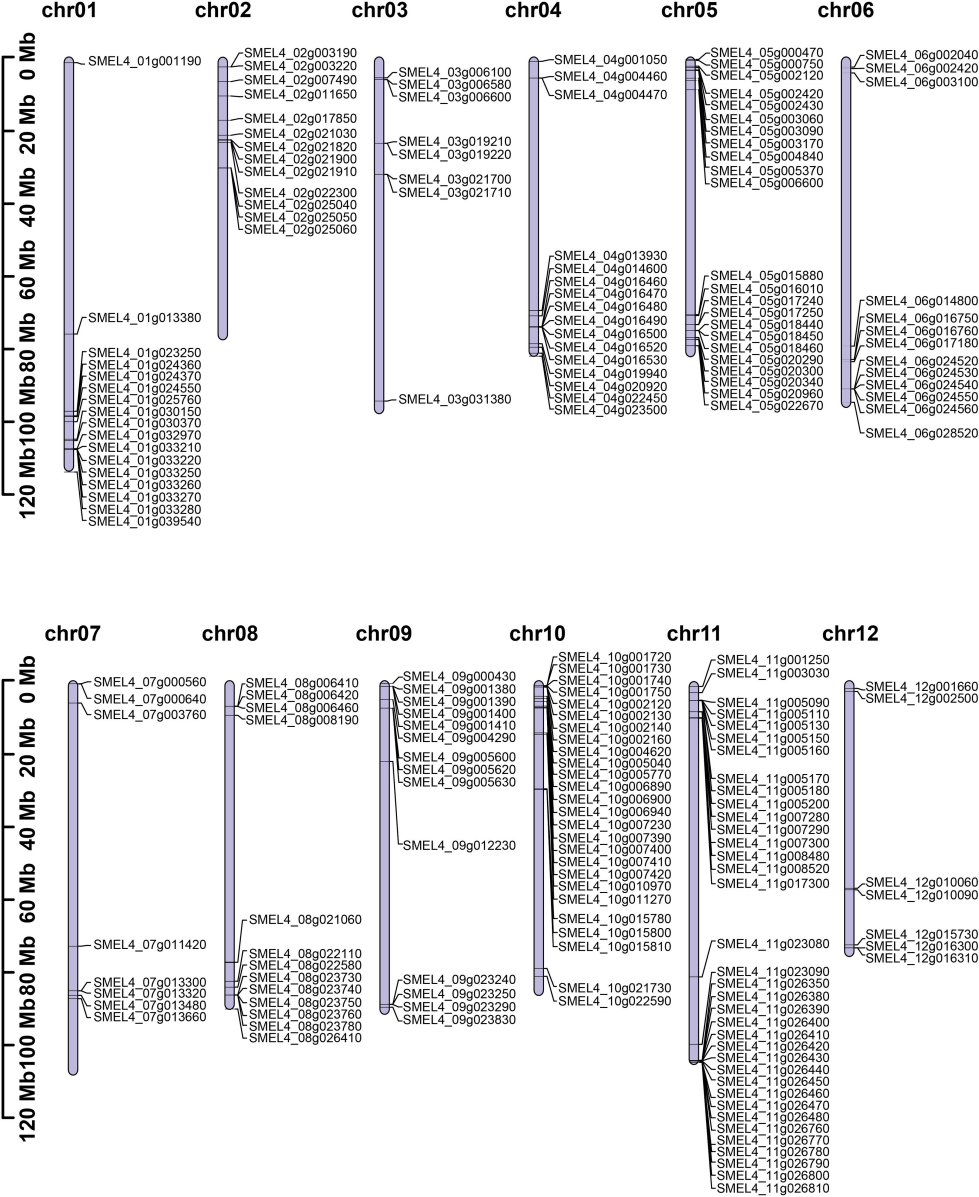
The distribution of chromosome locations. The cylinder represents the chromosomes, and the numbers above the column represent the number of chromosomes.

Based on the V4 version of the eggplant genome, the HMM model was used for searching *UGT* genes. After redundancy was removed by SMART and CDD-Search, a total of 195 *UGT* genes were identified. All candidate *UGT* genes contained a conserved UDPGT domain, which encoded amino acid sequences ranging from 154 aa to 1197 aa in length. The molecular weight (MW) of these proteins ranged from 17.02 kDa to 134.07 kDa, and the scope of the theoretical pI was 4.81 to 9.23. Most of the proteins were stable, and only fifty-seven (29% of the total) of SmUGT proteins were unstable (**Table S1**). Analysis of their chromosome location distribution showed that all candidate *UGT* genes were mapped on chromosomes 1-12, except for SMEL4_00g012200, which was not assembled to any chromosomes due to sequencing and assembly technology limitations. Among these chromosomes, chromosome 11 contained the largest number of SmUGT members (36). Chromosome 1, chromosome 5, and chromosome 10 contained 17, 23, and 26 members, respectively. Chromosome 12 (7) included the fewest number of SmUGT members (7) (**Figure 1**).

### Phylogenetic analysis of eggplant UGT family proteins

**Figure 2:**
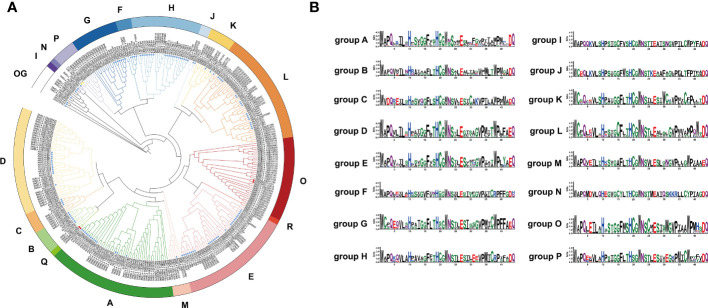
**(A)** Phylogenetic tree of the UGT family members. The tree consisted of all the identified *UGT* genes from eggplant, two *UGT* genes from rice (Li et al., 2014), four *UGT* genes from maize (Li et al., 2014; Huang et al., 2015) and all *Arabidopsis UGT* genes (Wilson and Tian, 2019). The tree was constructed using the maximum likelihood method. Different colored branches represent different groups. The blue star represents *Arabidopsis*. The yellow triangle represents rice, and the red circle represents maize. **(B)** Logos of the PSPG motif of phylogenetic Groups A - P generated by WebLogo (Crooks et al., 2004). The height of the amino acid indicates the frequency observed in multiple alignment.

To clarify the phylogenetic relationship of the UGT family in eggplant, a phylogenetic tree was constructed by the maximum likelihood method with all of the identified SmUGT protein sequences and those in other plants, including 122 *Arabidopsis UGTs*, two *UGT* genes of Group P in rice (Li et al., 2014) and four *UGT* genes of Groups Q and O in maize (Li et al., 2014; Huang et al., 2015). These UGT proteins from rice and maize were conducive to the classification of the SmUGT phylogenetic group. According to the phylogenetic tree, SmUGTS were clustered into 17 phylogenetic groups (A-P, R) and an outgroup (**Figure 2A**). As reported in *Arabidopsis*, *AtUGT84B1* from Group L was involved in glycosylation of IAA (Mateo-Bonmati et al., 2021). Both *AtUGT79B1* and *AtUGT91A1* from Group A were proven to mediate anthocyanin modification (Ono et al., 2010). *AtUGT89C1*, *AtUGT73C6* and *AtUGT78D1*, which belonged to Groups B, D and E, respectively, were related to glycosylation of flavonol (Jones et al., 2003; Yonekura-Sakakibara et al., 2007). It was likely that members of Groups A, B, D, E and L were related to phytohormone regulation and secondary metabolite biosynthesis.

To further understand the distribution of the UGTs across the phylogenetic range covered in different plant species, the number of UGT proteins distributed in phylogenetic groups is summarized in **Table 1**. Notably, some significant differences were observed in these phylogenetic groups. Compared with tomato, the members of eggplant UGTs were absent in Group Q, whereas the members of Solanaceae UGTs were present in Groups O, P and R compared with Arabidopsis, especially the members of Group R only found in Solanaceae. Group A contained the largest numbers of SmUGT members (35), followed by Groups O (30) and D (29). Groups F, I, J and N included a small number of UGT members in these plants. Compared with that of other species, the members of Group O in Solanaceae species had significant expansion, as well as tomato. The results indicated that Group O might play a vital role in Solanaceae plants.

**Table 1:** Summary of each group of UGT families in plant species.

Group	Eggplant	Tomato	*Arabidopsis*	Rice	Maize
A	35	24	14	14	8
B	5	2	4	9	3
C	4	2	4	8	5
D	29	17	13	26	18
E	17	16	25	38	34
F	2	2	4	/	2
G	11	13	7	20	12
H	6	6	21	7	9
I	1	2	1	9	9
J	2	1	2	3	3
K	8	5	2	1	1
L	25	20	18	23	23
M	5	3	1	5	3
N	1	1	1	2	4
O	30	25	/	6	5
P	7	6	/	9	1
Q	/	1	/	/	7
R	2	1	/	/	/
OG	5	15	5	/	/
Total	195	162	122	180	147

The PSPG-box was located in the UDPGT conserved domain. The PSPG box was distinct in different phylogenetic groups, in which highly conserved residues were observed at positions 1 (W), 4 (Q), 8 (L), 10 (H), 19-24 (HC/SGWNS), 27 (E), and 44 (Q) (**Figure 2B**). Some amino acids could interact directly with UDP-sugar donors to form hydrogen bonds, which were related to the evolution and function of enzymes. The amino acid residue Q (44) has been reported to determine the sugar donor specificity of UGTs, which affects the catalytic efficiency of glucosyl transfer activity (Ono et al., 2010; Caputi et al., 2012).

### Gene structure and conserved motif analysis of *SmUGTs*

**Figure 3:**
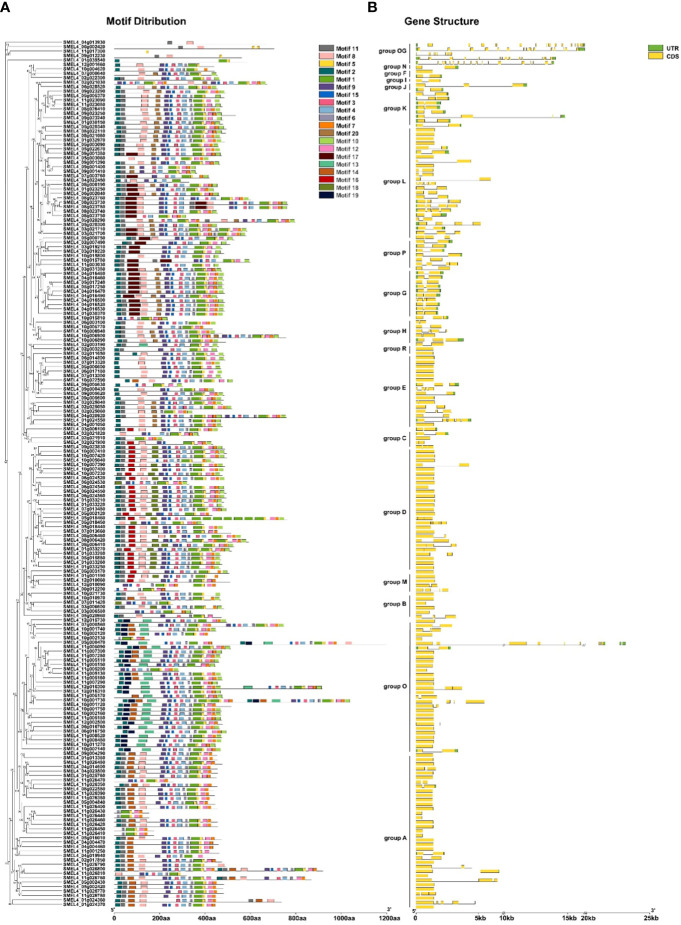
Conserved motifs and gene structure of *SmUGTs*. The phylogenetic tree was constructed by all *UGT* gene sequences using the maximum likelihood method, and the conserved motif **(A)** and gene structure **(B)** were placed orderly on the right side of the phylogenetic tree. Different colored boxes represent different motifs, and the box length represents the motif length in the motif distribution. The yellow box indicates exons; the green box indicates UTRs; and the black line indicates introns.

To investigate the structural features of SmUGT proteins, conserved amino acid sequences and gene structure were analyzed. Except for the outgroup, there were 89 members without introns (46%). The number of intron regions ranged from 1 to 7 in the rest of the members (**Figure 3B**). SMEL4_05g000470 (from Group O) was one of the most complex genes and had 7 introns. All members of Groups G, H, I, J, K, N, and P had introns. All SmUGT amino acid sequences were submitted to MEME, and a total of 20 conserved motifs were obtained (**Table S3**). These motifs possessed 11-50 residues and were named motif 1-motif 20. Motif 1, located in the PSPG box, existed in all SmUGT genes and served as a specific motif of the UGT family. In general, the same group has a similar motif distribution, whereas different groups possess a specific distribution of motifs. As shown in **Figure 3A**, motif 14 was mainly distributed in Groups O and A, while motif 16 and motif 18 were primarily present in Groups D and M. Motif 13 and motif 19 were found only in Group O.

### Expression analysis of *SmUGTs* in different developmental stages of eggplant fruit

**Figure 4:**
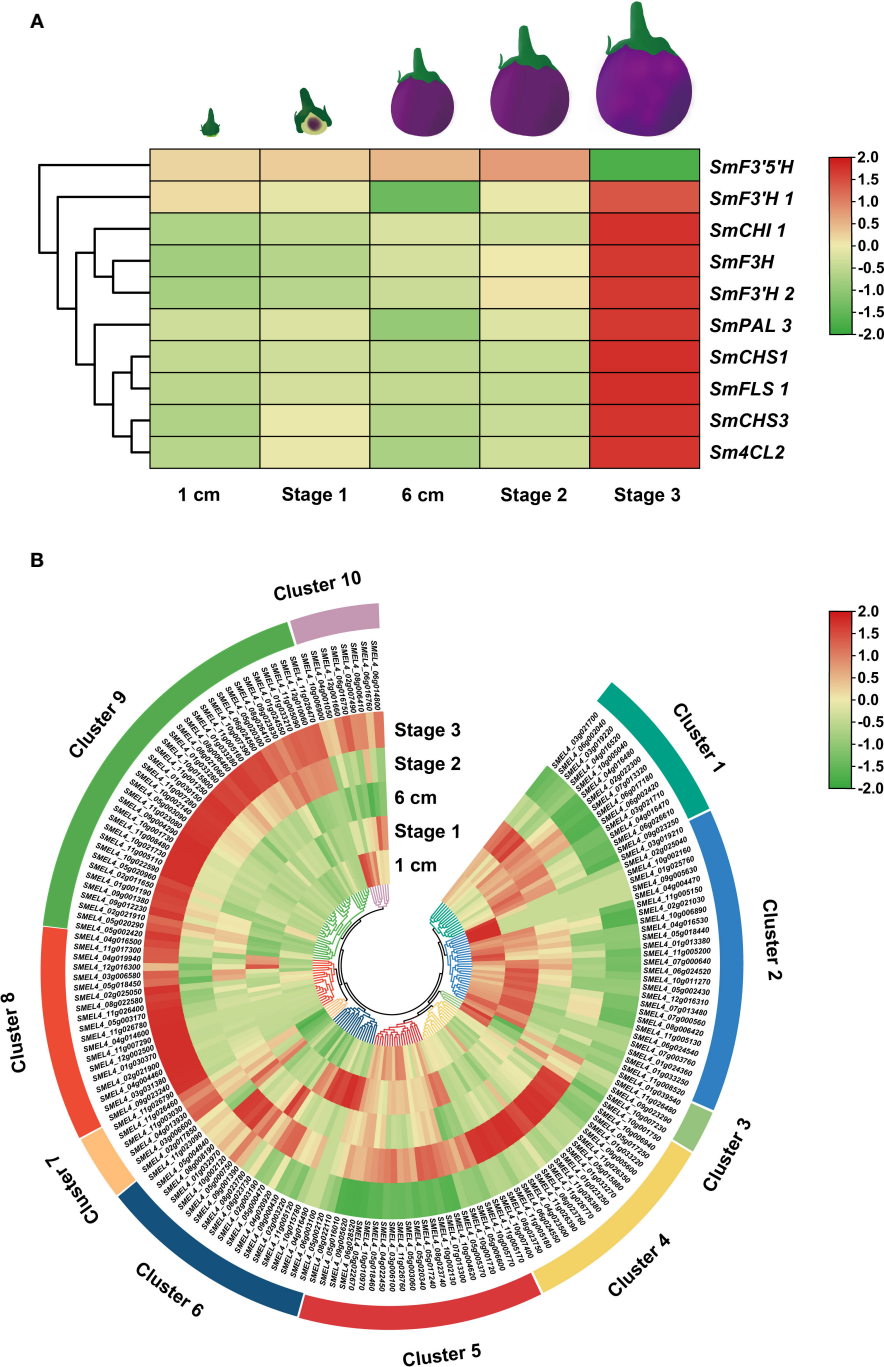
Expression patterns of rutin biosynthesis **(A)** and *UGT* **(B)** genes. Fruit 1 cm represents 3-4 days after flowering (DAF); fruit stage 1 represents 8-14 DAF; fruit 6 cm represents 18-21 DAF; fruit stage 2 represents approximately 38 DAF; fruit stage 3 represents 55-60 DAF.

Expression patterns can predict the biological functions of genes to a certain extent. To investigate the underlying functions of candidate *UGT* genes during rutin biosynthesis processes, transcriptome data of eggplant at different developmental stages from a public database were downloaded (Barchi et al., 2019). The expression patterns of candidate *SmUGTs* and rutin biosynthesis genes in different developmental stages of eggplant fruit were analyzed. The rutin biosynthesis genes were highly expressed in stage 3 (**Figure 4A**). A total of 23 *SmUGTs* (12%) were expressed at a level that could not be detected. Based on the similar expression trend, all *SmUGTs* in different developmental fruit stages were hierarchically clustered and divided into 10 clusters (**Figure 4B**). The expression patterns of Clusters 7, 8, 9 and 10 were similar to the expression patterns of rutin biosynthesis genes, and their expression levels gradually increased with fruit development. Therefore, the members of Clusters 7–10 served as novel candidates for the regulation of rutin biosynthesis.

### Expression patterns of *SmUGTs* in different tissues and different developmental stages of fruits and leaves in eggplant

**Figure 5:**
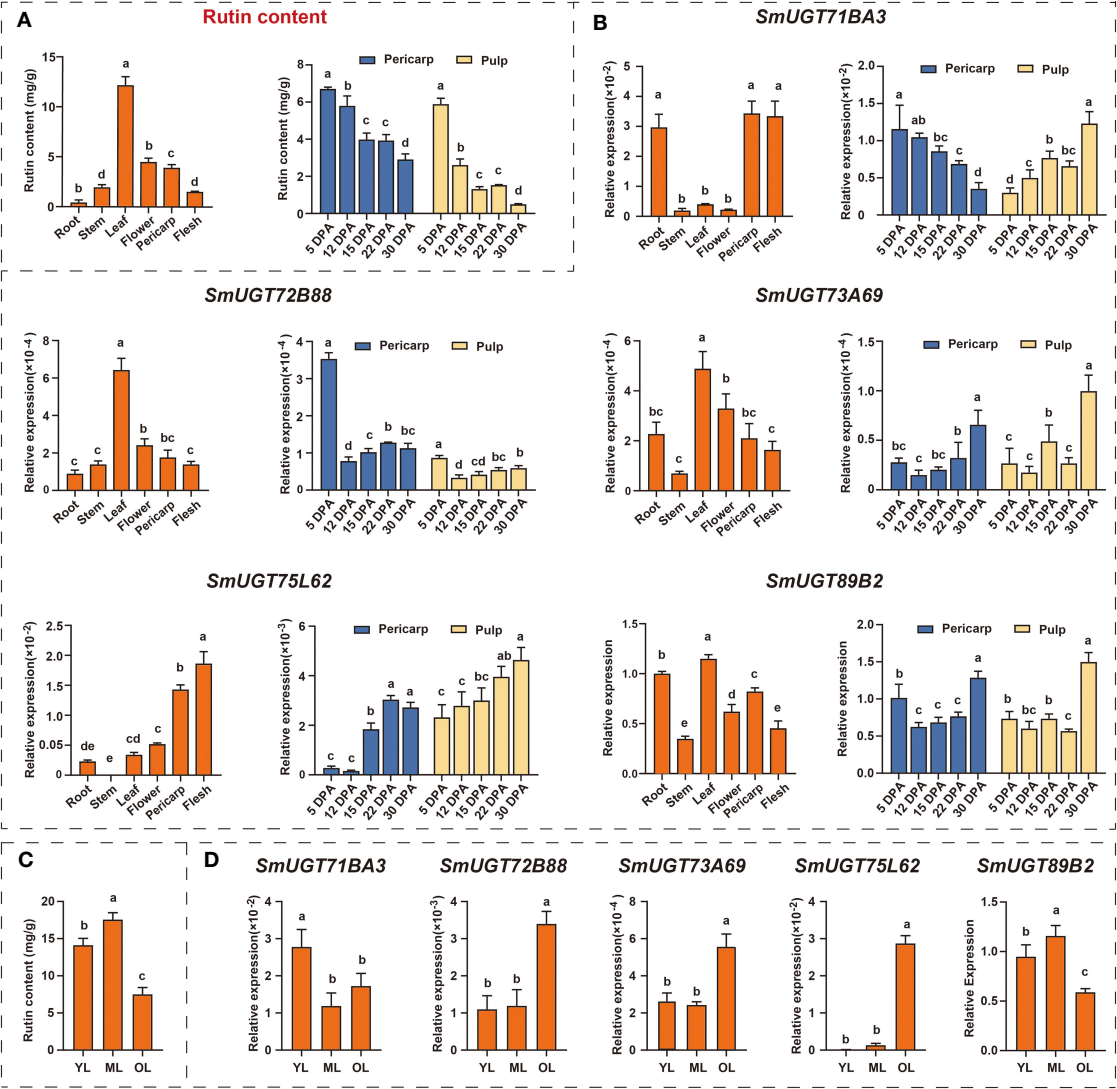
The expression patterns of five *SmUGT* genes in different tissues and different developmental stages of leaves and fruit in eggplant (E801). **(A)** Rutin content in different eggplant tissues (roots, stems, leaves, flowers and pericarp and pulp). **(B)** Expression pattern of five *SmUGT* genes in different eggplant tissues (roots, stems, leaves, flowers, pericarp and flesh). Data are the mean ± SE (n = 3). Different lowercase letters in the figures represent significantly different values (P < 0.05, Tukey’s test). **(C)** The rutin content at different developmental stages of leaves in E801. **(D)** Expression of five *SmUGT* genes at different developmental stages of leaves. Different lowercase letters in the figures represent significantly different values (P < 0.05, Tukey’s test).

To further identify *SmUGT* genes involved in rutin biosynthesis in eggplant, five *SmUGT* genes that belonged to Groups A-F and L with a higher expression level were selected from candidate Clusters 7 to 10 to perform qRT−PCR assays. These five genes were renamed by the UGT Nomenclature Committee, called *SmUGT71BA3*, *SmUGT72B88*, *SmUGT73A69*, *SmUGT75L62* and *SmUGT89B2*, respectively (**Table S2**). Rutin accumulated in all tissues of eggplant, and the highest content was found in leaves, followed by flowers and pericarp. Meanwhile, the highest content was found at 5 DPA, and with the development of fruit, the rutin content gradually decreased (**Figure 5A**). The expression of *SmUGT72B88*, *SmUGT73A69* and *SmSmUGT89B2* was the highest in leaves, followed by flowers and fruits, which was consistent with the accumulation of rutin in different tissues of eggplant (**Figure 5B**). However, *SmUGT71BA3* was highly expressed in roots and fruits compared with that in other tissues. The expression of *SmUGT75L62* was highest in pericarp, whereas it was transcribed at a low level in stem that was not detectable. The expression pattern of *SmUGT71BA3* was inversely represented in pericarp and pulp, showing a significant decrease in pericarp and a marked increase in pulp from 5 DPA to 30 DPA. In the other four genes, including *SmUGT72B88*, *SmUGT73A69*, *SmUGT75L62* and *SmUGT89B2*, their own expression levels were similar in the pericarp and pulp. The expression levels of *SmUGT73A69* and *SmUGT75L62* exhibited a growing tendency with the process of fruit development, while *SmUGT72B88* and *SmUGT89B2* were observed to have higher expression at 5 DPA and then showed a decrease at 12 DPA. Their expression levels were not significantly different from 12 DPA to 30 DPA, except for that of *SmUGT89B2*, which increased at 30 DPA (**Figure 5B**).

To further explore the expression patterns of these five genes, leaves at different developmental stages were used for verification. During the course of leaf senescence, the rutin content gradually increased from young leaves to mature leaves and then decreased, with a peak at mature leaves (**Figure 5C**). As shown in **Figure 5D**, the transcription levels of *SmUGT72B88, SmUGT73A69* and *SmUGT75L62* increased with the process of leaf senescence. The expression patterns of *SmUGT89B2* maintained good agreement with the accumulation of rutin in different developing leaves.

### Sequence analysis and subcellular location of *SmUGT89B2*

**Figure 6:**
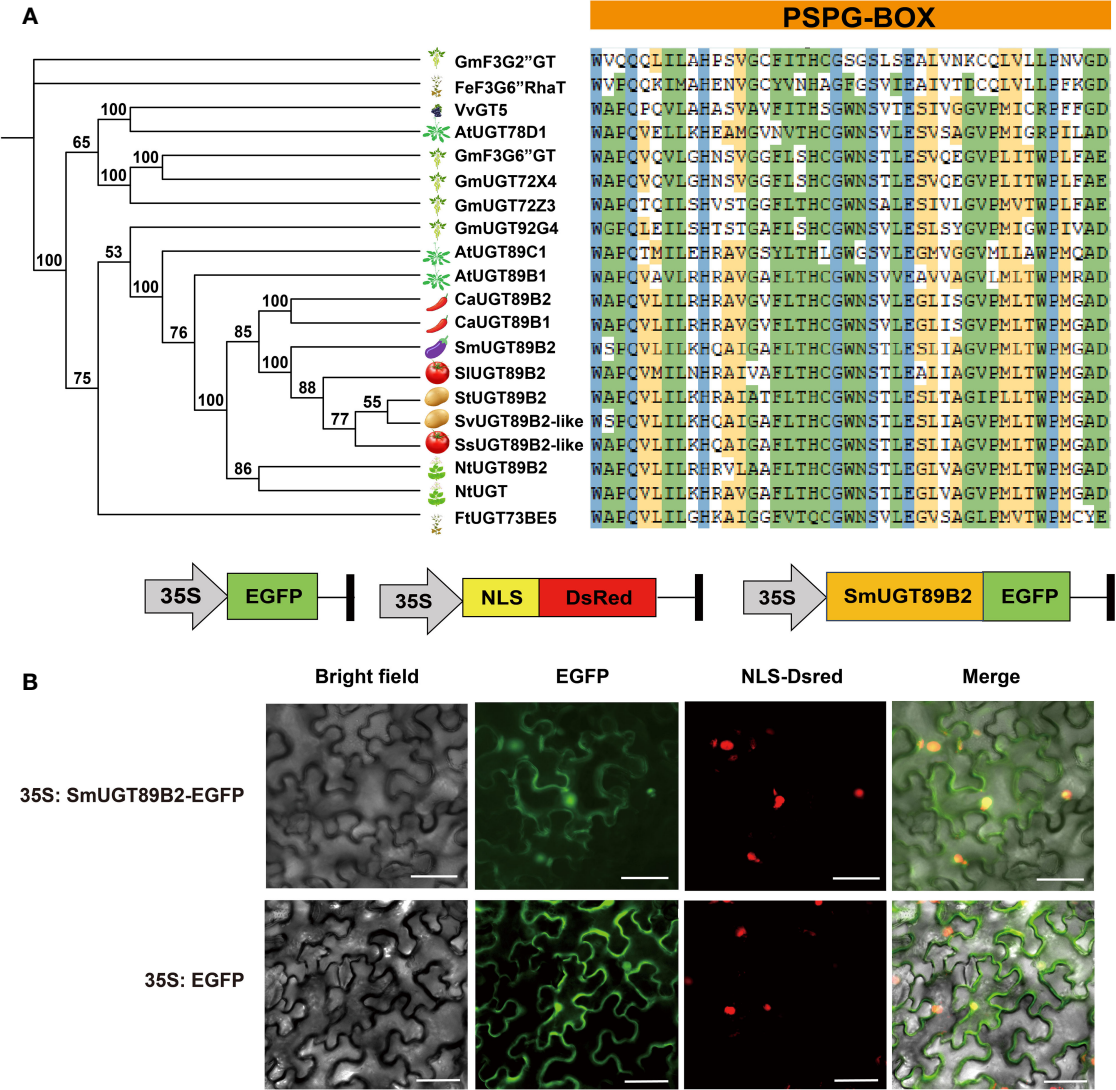
Sequence analysis and subcellular localization of SmUGT89B2. **(A)** Multiple alignment of PSPG-box motif (right) and a phylogenetic tree (left) of *SmUGT89B2* with homologous genes from other plant species. The sequences of other plant species were obtained from NCBI and soybean database. A phylogenetic tree was built *via* MEGA X using the maximum likelihood method with 2000 bootstrap replicates. **(B)** Subcellular localization of SmUGT89B2. EGFP represents enhanced GFP. NLS-DsRed served as a nuclear marker, and fluorescence was visualized using an inverted fluorescence microscope. Scale bar = 50 μm.

To determine whether *SmUGT89B2* is involved in rutin biosynthesis in eggplant, the amino acid sequence of SmUGT89B2 was analyzed. The full-length CDS of *SmUGT89B2* was cloned from the E801 inbred line. Its protein encoded 483 amino acids. The molecular weight (MW) of SmUGT89B2 was 53.83 kDa, and theoretical PI was 6.44. It is an unstable protein, and the instability index was 42.04. The aliphatic index (95.51) was predicted that SmUGT89B2 was a hydrophilic protein (**Table S1**). Several UGT proteins that have been reported to be involved in flavonol glycosylation were obtained (Carbonell-Bejerano et al., 2014; Yin et al., 2017; Koja et al., 2018; Yin et al., 2020), and some UGT proteins from other Solanaceae species that shared the highest sequence identity with SmUGT89B2 were retrieved from public databases (NCBI, https://www.ncbi.nlm.nih.gov/). All of them were used to construct an unrooted phylogenetic tree based on their multiple sequence alignment. As shown in the phylogenetic tree, the UGT genes involved in flavonol glycosylation in the different species had a distant evolutionary relationship with SmUGT89B2. The conserved PSPG-box motif existed in all UGT amino acid sequences, in which some amino acids were highly conserved (**Figure 6A**) (Caputi et al., 2012). To clarify the subcellular localization of SmUGT89B2, its sequence was fused in frame with the EGFP gene to generate the 35S:SmUGT89B2-EGFP construct (**Figure 6B**). The DsRed gene served as the nuclear marker by fusion with a nuclear localization signal (NLS) (Sun et al., 2020). The coexpression of either 35S: EGFP or 35S:SmUGT89B2-EGFP with DsRed in epidermal cells of *Nicotiana benthamiana* leaves indicated that SmUGT89B2 was localized in the nucleus and cell membrane, as well as in the cytoplasm (**Figure 6B**). It was likely that SmUGT89B2 functioned in the whole cell.

### *SmUGT89B* can positively promote rutin accumulation in eggplant

**Figure 7:**
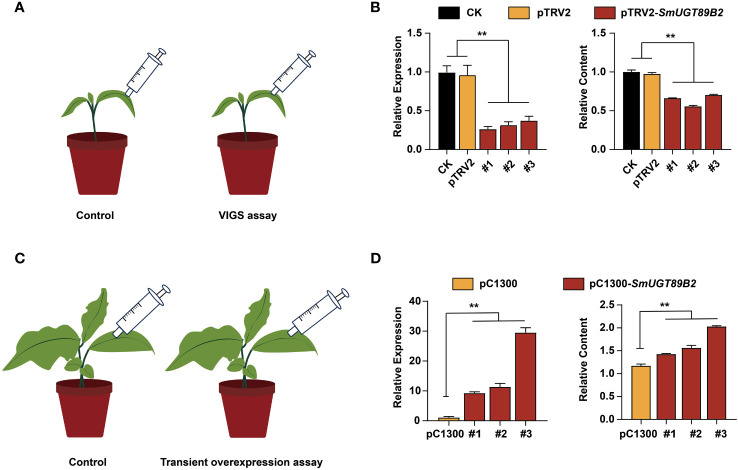
Silencing and transient overexpression of *SmUGT89B2* in eggplant. **(A)** Schematic diagram of the SmUGT89B VIGS assay. **(B)** The expression of *SmUGT89B* and relative rutin content in leaves of *SmUGT89B*-silenced eggplants. CK were injected with water. pTRV2 was injected with *Agrobacterium* harboring an empty vector. Data are the mean ± SE (n = 3). **(C)** Schematic diagram of the transient overexpression assay for *SmUGT89B2*. **(D)** The expression of *SmUGT89B* and relative rutin content in leaves of *SmUGT89B*-overexpressed eggplants. pC1300 were the plants whose leaves were injected with *Agrobacterium* harboring an empty vector. mean ± SE (n = 3). Student’s t test was used to indicate significant differences compared to those in the empty vector control (**P < 0.01).

To determine whether *SmUGT89B2* was associated with rutin accumulation in eggplant, the transcriptional level of *SmUGT89B2* was silenced in the E801 inbred line through virus-induced gene silencing (VIGS). Three silenced eggplant lines that possessed the highest silencing efficiency were selected to determine *SmUGT89B2* expression and the rutin content. As shown in **Figure 7B**, the expression level of *SmUGT89B2* was significantly decreased in silenced lines, reducing by approximately 60% of that in the control plants. Simultaneously, the rutin content in the silenced leaves decreased by approximately 40% of that in the control leaves. To further confirm whether *SmUGT89B2* plays a role in rutin biosynthesis in eggplant, a transiently overexpressed assay was performed in eggplant seedlings at the five-leaf stage. The *SmUGT89B2* transcription levels increased 8.8-28-fold in the overexpressed leaf compared with those in the control, consistent with the 1.2-1.7-fold increase in rutin content in overexpressed leaf (**Figure 7D**). These results suggested that *SmUGT89B2* played an important role in rutin biosynthesis in eggplant.

## Discussion

Glycosyltransferase is a supergene family, and plant *UGTs* are classified into the first family (GT1) of the glycosyltransferase (GT) family. Glycosylated metabolites generated by UDP-dependent glycosyltransferases play important roles in plant interactions with the environment as well as human and animal nutrition (Wilson and Tian, 2019). The UGT family has been identified in various plants, including *Arabidopsis* (123) (Li et al., 2001), cotton (274) (Xiao et al., 2019), maize (147) (Li et al., 2014), peach (168) (Wu et al., 2017), buckwheat (106) (Yin et al., 2020), soybean (212) (Yin et al., 2017), tea (178) (Cui et al., 2016), pomelo (145) (Wu et al., 2020), and *Brassica rapa* (147) (Yu et al., 2017). The numbers of *UGT* genes present significant differences between various plants. In this study, a total of 195 *UGT* genes identified from the eggplant genome were divided into 17 groups by constructing a phylogenetic tree with other plant *UGTs*, containing 14 highly conserved phylogenetic groups (A-N) and three new phylogenetic groups (O, P, and R) (**Figure 2A**). Group O contained 30 *UGT* members in eggplant, while it contained approximately the same amount of UGT members in tomato (25) (Wilson and Tian, 2019) and tabacoo (40) (Yang et al., 2023). In contrast, the number of *UGT* members was dramatically reduced in rice (6), maize (5) and other dicotyledon plants (Wilson and Tian, 2019). Thus, the members of Group O were significantly expanded in Solanaceae plants and seem to be unique to Solanaceae species. *UGT709G2* from Group P shows a substrate preference for UDP-Glc and catalyzes myricetin 3-O-rhamnoside into myricetin 3-O-glucosyl rhamnoside in montbretia (Irmisch et al., 2018). The members of Group R were recently discovered and first identified in buckwheat. *FeUGT708C1* from Group R could recognize and catalyze the C-glycosylation of trihydroxy acetophenone-like compounds (Nagatomo et al., 2014). It appears that the members of new phylogenetic groups are related to the glycosylation of secondary compounds in different plants. Group R was absent in all Brassicales but present in Solanaceae species. which was probably responsible for the distant relationship between them. According to the phylogenetic evolutionary tree, five *SmUGT* genes with two *Arabidopsis* genes (*AtUGT80A2* and *AtUGT80B1*) were classified into a separate out group (OG) (**Figure 2A**), in which the PSPG-box was less conserved. Members of the OG group have been proposed to modify sterols and lipids in other plants (Paquette et al., 2003). It is likely that the five genes may be involved in the formation of sterols and lipids in eggplant.

The members defined in the same phylogenetic subgroup generally possess similar functions (Soler et al., 2015; Song et al., 2020). Some *UGT* genes belonging to Groups B, D, E and L have been identified. *AtUGT73C6* (Group D) can catalyze the transfer of glucose from UDP-glucose to kaempferol-3-O-rhamnoside and quercetin-3-O-rhamnoside (Jones et al., 2003). *FaGT6*, which belongs to the UGT71 family, catalyzes quercetin into 3-O-glucoside (Griesser et al., 2008). UGT75 family members are mostly flavonoid-5-O-glycosyltransferases, which are mainly involved in the synthesis of plant anthocyanins (Noguchi et al., 2009; He et al., 2015). Therefore, the members from Groups B, D, E and L might possess the underlying function of participating in flavonol biosynthesis.

Based on the sequence analysis of a multigene family, its gene expression compared with that in the expression profiles of known genes and complementation of the metabolite pattern in combination with reverse genetics are conducive to understanding the underlying function of a single member from the superfamily (Offen et al., 2006). The expression patterns of SmUGT members from Clusters 7 to 10 exhibited good agreement with the transcription levels of rutin biosynthetic genes in eggplant (**Figure 4B**). Thus, the members of Clusters 7 to 10 acted as novel candidates for rutin biosynthesis. *SmUGT89B2* found in Cluster 7 was classified into Group B, as well as *AtUGT89C1*. *AtUGT89C1* has been proposed to be involved in flavonol biosynthesis in *Arabidopsis* (Yonekura-Sakakibara et al., 2007). It seems that SmUGT89B2 may be involved in flavonol biosynthesis in eggplant. The other four UGT genes observed in RNA-Seq data with high RPKM values, including *SmUGT71BA3* and *SmUGT72B88* (Group E), *SmUGT73A69* from Group D and *SmUGT75L62* from Group L, which were found in Cluster 9, were selected for qRT−PCR assays, as well as *SmUGT89B2* (**Figure 4B**).

The expression pattern of these five genes was unlike the rutin accumulation pattern at different developmental stages of fruit in eggplant (**Figure 5A**). The rutin content was the highest at the young fruit stage. It seems that plants can activate self-protection processes via the production of secondary metabolites against undesirable circumstances, such as UV irradiation, cold stress, and desiccation stress (Suzuki et al., 2005; Yang et al., 2012). This defense reaction was previously reported in anthocyanin accumulation (Naing and Kim, 2021). A large number of secondary metabolites are needed to protect plants, especially in tender tissues. The expression of *SmUGT89B2* was similar to the accumulation pattern of rutin in eggplant (**Figure 5**). Therefore, *SmUGT89B2* served as a novel candidate gene for rutin biosynthesis in this study. The subcellular localization results showed that SmUGT89B2 was localized in the nucleus and cell membrane, as well as in the cytoplasm (**Figure 6B**). Most *UGT* genes have multiple subcellular localizations to control where specific pathway products accumulate (Yi et al., 2020; Daryanavard et al., 2023). Transient overexpression and silencing expression of *SmUGT89B2* in eggplant indicated that *SmUGT89B2* could affect rutin accumulation (**Figure 7**). It was likely that SmUGT89B2 encoded the final enzyme in the rutin biosynthetic pathway. Although the functions of UGT89B2 involved in rutin biosynthesis in eggplants *in vivo* have been identified in this study, the enzyme activity and biochemical characteristics of UGT89B2 should be further identified. Which step of SmUGT89B2 involved in two glycosylation steps of quercetin transform to rutin also need to be verified by catalytic experiments. But the exploration of this catalytic experiment is certainly the most difficult because UGT glycosyltransferase possesses substrate diversity. In the future, enzymatic tests of the SmUGT89B2 will be the key works for our studies.

## Conclusion

Overall, all *UGT* genes from the eggplant genome through the HMM model and PSPG-box motif were searched in the study. A total of 195 *SmUGT* genes were identified, which were divided into 17 phylogenetic subgroups according to the classification of *Arabidopsis*, maize and rice. The conserved motifs and gene structures of candidate *UGT* genes were analyzed. According to the expression profile analysis, Clusters 7-10 served as candidate clusters, in which the five genes *SmUGT71BA3*, *SmUGT72B88*, *SmUGT73A69*, *SmUGT75L62* and *SmUGT89B2* were selected as candidate genes. However, among these selected genes, *SmUGT89B* had a higher transcription level in eggplant, and its expression pattern was similar to the accumulation trend in eggplant by qRT−PCR assay. VIGS and transient overexpression demonstrated that *SmUGT89B2* could positively promote rutin biosynthesis in eggplant.

## Data availability statement

The datasets presented in this study can be found in online repositories. The names of the repository/repositories and accession number(s) can be found in the article/**Supplementary Material**.

## Author contributions

YG: Data curation, Formal analysis, Writing – original draft, Visualization, Writing – review & editing. BY: Writing – original draft. RL: Formal analysis, Visualization, Writing – original draft. BS: Data curation, Validation, Writing – original draft. YL: Data curation, Validation, Writing – original draft, Formal analysis. YZ: Writing – original draft. ZQ: Data curation, Project administration, Writing – original draft. SY: Project administration, Writing – original draft, Writing – review & editing. BC: Funding acquisition, Project administration, Writing – original draft, Writing – review & editing.
