# Supplementary material for: Systematic analysis of the UDP-glucosyltransferase family: discovery of a member involved in rutin biosynthesis in Solanum melongena
Source: Front Plant Sci. 2023 Dec 22;14:1310080. doi: 10.3389/fpls.2023.1310080 (PMC10774229; doi:10.3389/fpls.2023.1310080)
Supplement: Supplementary file 1 [file DataSheet_1.docx]

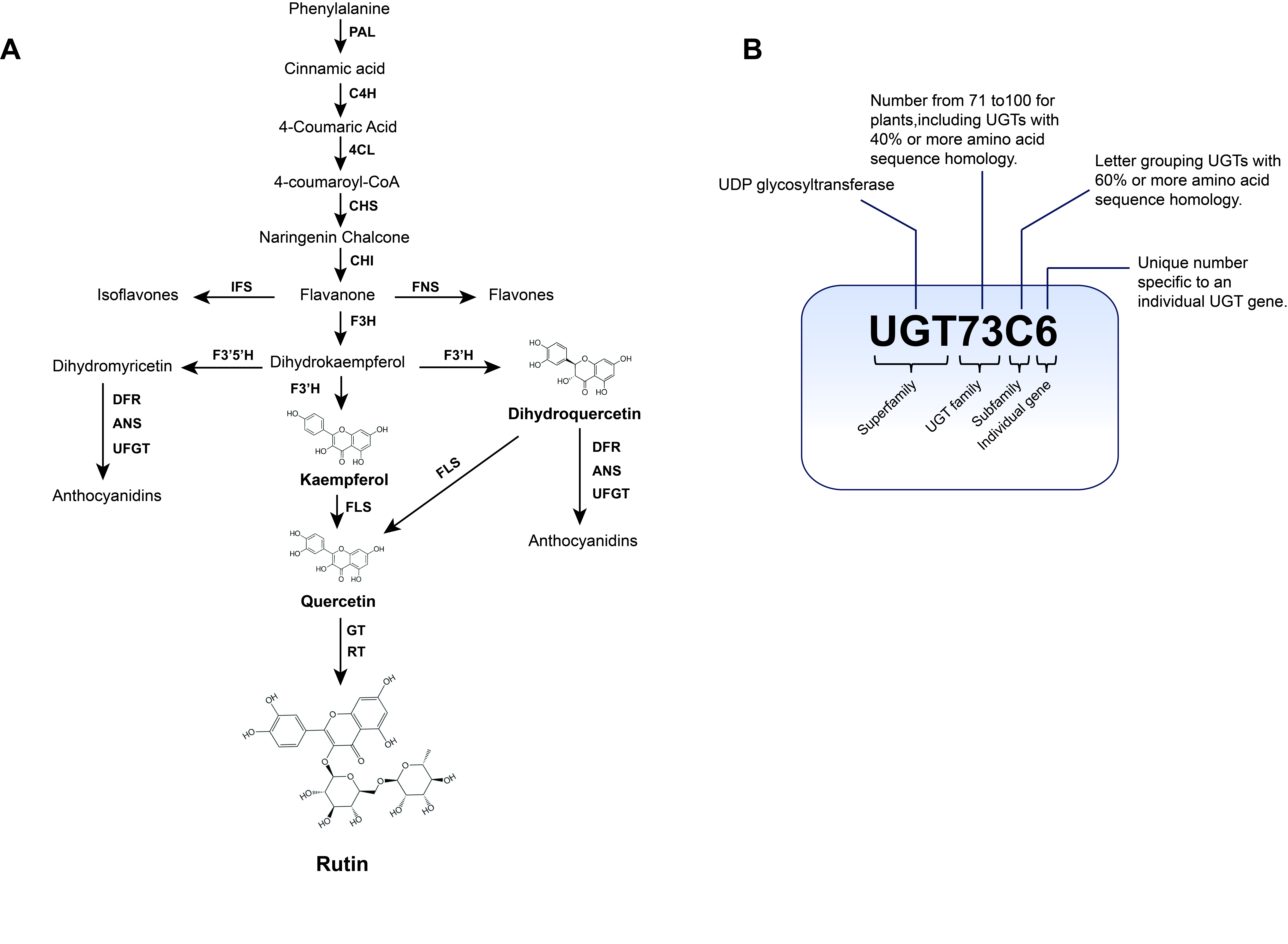


Supplementary Figure 1. (A)The rutin biosynthetic pathway (Zhang et al., 2017; Shen et al., 2022; Koja et al., 2018). (B)The nomenclature system for UGT genes. The root sign UGT, the family denoted by an Arabic number, the subfamily represented by a letter, and the individual gene indicated by an Arabic number at the end of the name. UGT genes are name by the UGT Nomenclature Committee(https://labs.wsu.edu/ugt/).


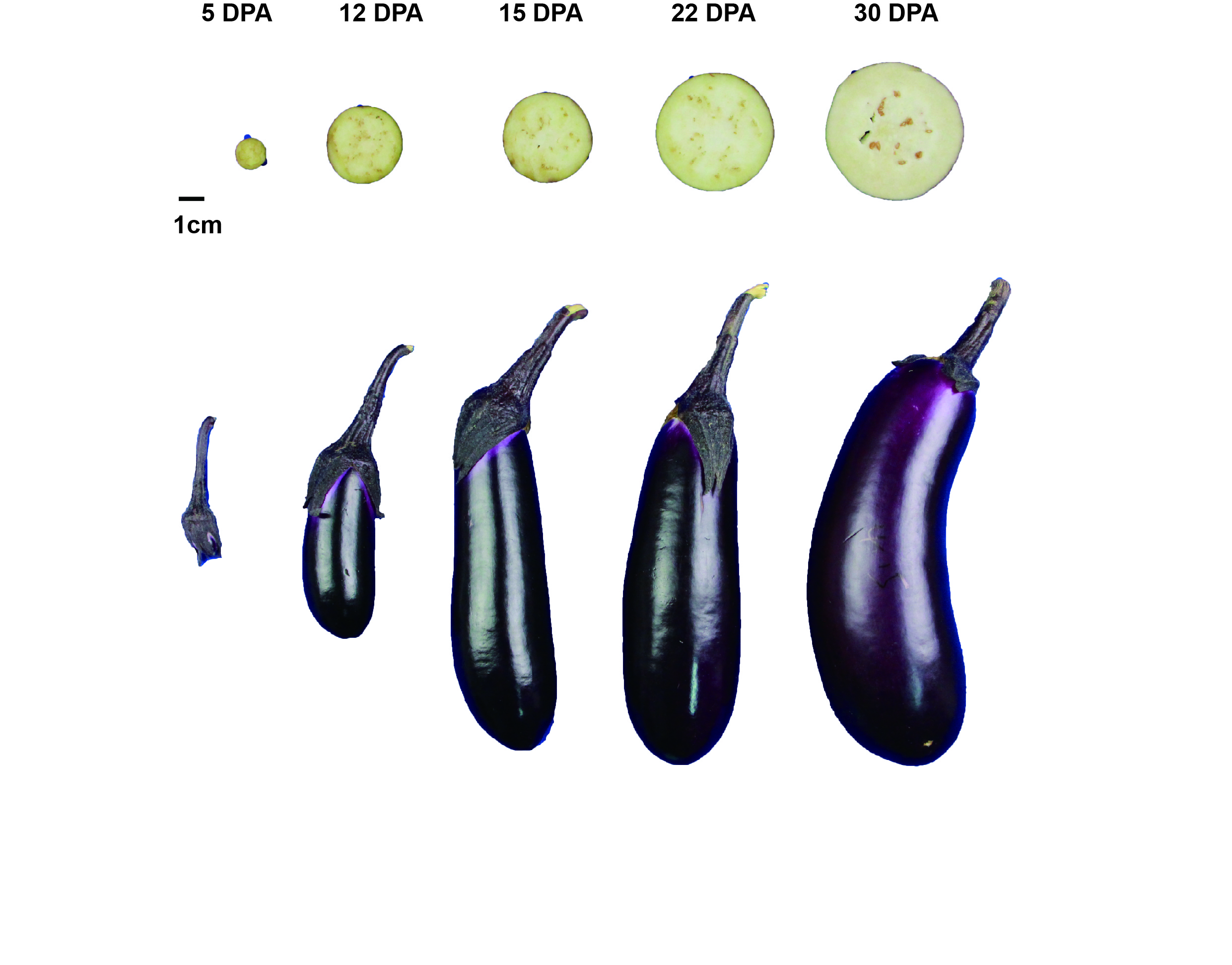


Supplementary Figure 2. Eggplant fruits at 5 different developmental stages. Fruits Ø at 5 DPA, with the fruit still almost fully covered by sepals; fruits Ø 3cm at 12 DPA; fruits Ø 5-6 cm at 15 DPA; fruits Ø 6-7 cm at 22 DPA (commercial ripening); fruits Ø 7-8 cm at 30 DPA (physiological ripening). Scale bar represents 1cm.
