# Supplementary material for: Systematic analysis of the UDP-glucosyltransferase family: discovery of a member involved in rutin biosynthesis in Solanum melongena
Source: Front Plant Sci. 2023 Dec 22;14:1310080. doi: 10.3389/fpls.2023.1310080 (PMC10774229; doi:10.3389/fpls.2023.1310080)
Supplement: Supplementary file 4 [file Table_3.pdf]

Table S3. Basic information of conserved motifs of 195 candidate SmUGT protein sequences

Motif	Sequence of conserved domains	E-value	Sites	Width	logo
1	PQLEILSHPSIGGFLTHCGWNSTLESISFGVPMIAWP	1.3e-4969	190	37	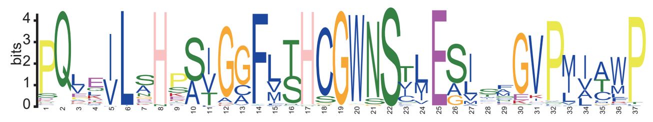
2	KLHVVMVPFPAQGHJNPLLQL	1.4e-1419	165	21	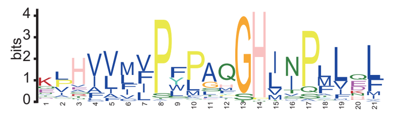
3	WLDKQPPNSVVYVSF	1.5e-1379	177	15	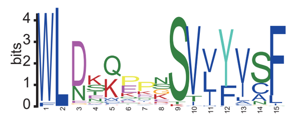
4	QLEEJALGLENSGVPFJWVIR	5.4e-1748	174	21	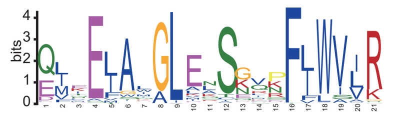
5	MFADQPTNAKL	8.3e-656	195	11	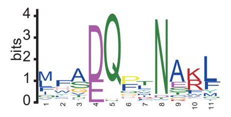
6	EGFEERVKGRG	3.9e-527	146	11	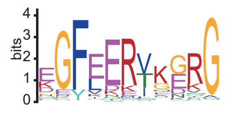
7	GEEGEEIRKRAKELK	1.9e-610	170	15	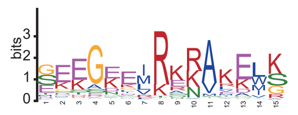
8	PPPDCJISDMFLPWTVDVAKELGIPRVVF	1.2e-1233	139	29	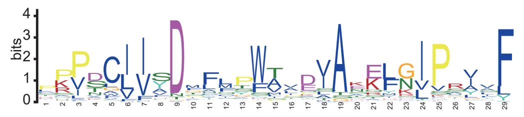
9	KADGIJINTFRELEGEYLDYL	8.7e-914	175	21	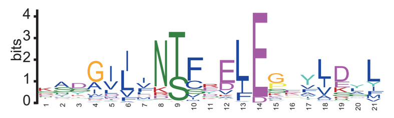
10	KNAVEEGGSSYKNLDSFIDDI	3.6e-756	133	21	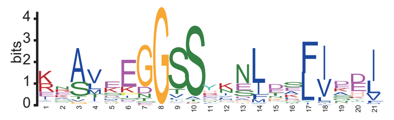
11	AKLLASKGVKITFITTPLNAK	2.2e-648	125	21	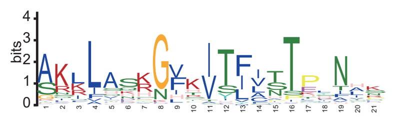
12	EDEDGLVKREEIEKAVRELMV	2.1e-701	163	21	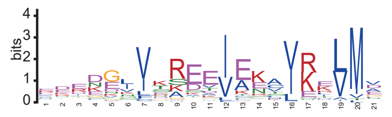
13	SSKSRRVVVVHDSLMSYVVQDVSSJPNAESYIFHSISAF	1.5e-490	26	39	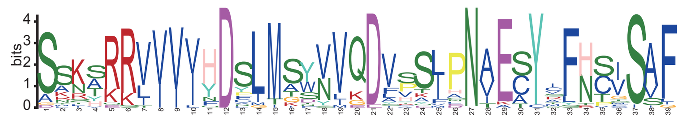
14	SIHFVEIPLPEVPGLPPHAHTTNDLPPHL	7.6e-385	39	29	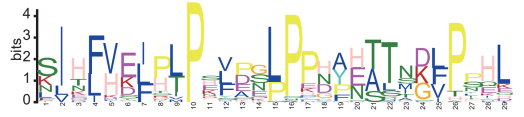
15	PVWAIGPLLPS	1.1e-353	189	11	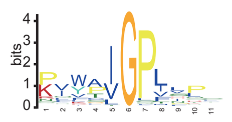
16	ISIREJKFPSVEVGLPEGCENFDSLPSPE	6.60E-298	31	29	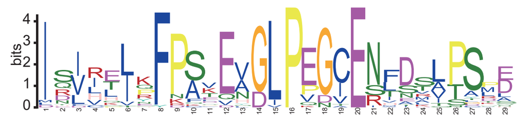
17	GLPSFRFETIPDGLPPSDADATQDIPSLCESTKETCLDPFRZLLAKLNBT	5.20E-250	17	50	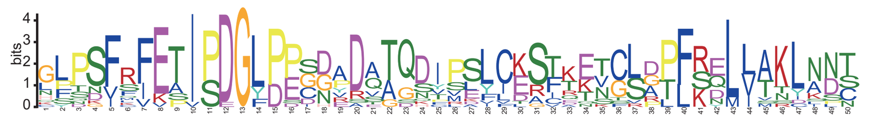
18	KKVISDSESFVVPGLPDKIEMTKSQLPGA	3.80E-221	26	29	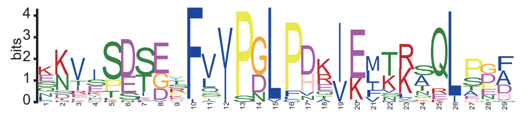
19	ISSYNJPVHYVGLATHNRQAKVRANALNP	2.80E-204	21	29	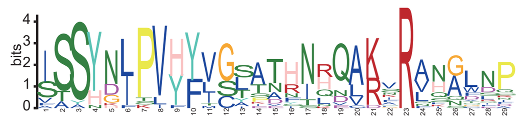
20	LDYIPGMKPJRLKDLPSFLRD	5.90E-196	42	21	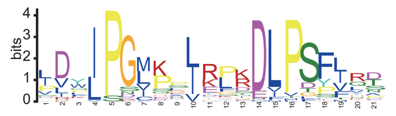
